# MRI findings in men on active surveillance for prostate cancer: does dutasteride make MRI visible lesions less conspicuous? Results from a placebo-controlled, randomised clinical trial

**DOI:** 10.1007/s00330-017-4858-0

**Published:** 2017-05-18

**Authors:** Francesco Giganti, Caroline M. Moore, Nicola L. Robertson, Neil McCartan, Charles Jameson, Simon R. J. Bott, Mathias Winkler, Giulio Gambarota, Brandon Whitcher, Ramiro Castro, Mark Emberton, Clare Allen, Alex Kirkham

**Affiliations:** 1 Department of Radiology, University College London Hospital NHS Foundation Trust, 235 Euston Road, London, NW1 2BU UK; 2 Division of Surgery & Interventional Science, University College London, London, UK; 3 Department of Urology, University College London Hospital NHS Foundation Trust, London, UK; 4 Department of Pathology, University College London Hospital NHS Foundation Trust, London, UK; 5 Department of Urology, Frimley Park Hospital, Surrey, UK; 6 Department of Urology, Charing Cross Hospital, Imperial College NHS Trust, London, UK; 7 INSERM, U1099, Rennes, F-35000 France; 8 Université de Rennes 1, LTSI, Rennes, F-35000 France; 9 Klarismo, London, UK; 10 Department of Mathematics, Imperial College London, London, UK; 11 Research and Development, GlaxoSmithKline, Philadelphia, PA USA

**Keywords:** Prostatic neoplasms, Diffusion Magnetic Resonance Imaging, Molecular Imaging, Dutasteride, Placebo

## Abstract

**Objectives:**

To investigate changes in the Apparent Diffusion Coefficient (ADC) using diffusion-weighted imaging (DWI) in men on active surveillance for prostate cancer taking *dutasteride* 0.5 mg or *placebo*.

**Methods:**

We analysed 37 men, randomised to 6 months of daily dutasteride (n = 18) or placebo (n = 19), undergoing 3T multi-parametric Magnetic Resonance Imaging (mpMRI) scans at baseline and 6 months. Images were reviewed blind to treatment allocation and clinical information. Mean ADC of peripheral (PZ) and transition (TZ) zones, and MR-suspicious lesions were compared between groups over 6 months. Conspicuity was defined as the PZ divided by tumour ADC, and its change over 6 months was assessed.

**Results:**

A decrease in mean *conspicuity* in the dutasteride group (but not the controls) was seen over 6 months (1.54 *vs* 1.38; p = 0.025). Absolute changes in ADC and *conspicuity* were significantly different between placebo and dutasteride groups at 6 months: (-0.03 *vs* 0.08, p = 0.033) and (0.11 *vs* –0.16, p = 0.012), as were percentage changes in the same parameters: (-2.27% vs 8.56% p = 0.048) and (9.25% vs -9.89% p = 0.013).

**Conclusions:**

Dutasteride was associated with increased tumour ADC and reduced *conspicuity*. A lower threshold for triggering biopsy might be considered in men on dutasteride undergoing mpMRI for prostate cancer.

***Key points*:**

• *Dutasteride increases ADC and reduces conspicuity in small mpMRI*-*visible prostate cancers*.

• *Knowledge of dutasteride exposure is important in the interpretation of prostate mpMRI*.

• *A lower threshold for triggering biopsy may be appropriate on dutasteride*.

## Introduction

Prostate cancer often behaves in an indolent fashion even without treatment, so that many men with small low-grade tumours are suitable for active surveillance.

Dutasteride inhibits the enzyme 5 alpha-reductase that converts testosterone to dihydrotestosterone (DHT), and is widely used for the treatment of lower urinary tract symptoms (LUTS) associated with an enlarged prostate [1]. Its clinical use for prostate cancer is not licensed but has been investigated in four published studies.

The first was a large randomised study in 6729 men (REDUCE) by Andriole et al. that showed that dutasteride reduced the period prevalence of prostate cancer by 24% compared to placebo [2]. The second study (REDEEM) randomised men with low risk prostate cancer on active surveillance to daily dutasteride 0.5 mg or placebo over 3 years. Fifty-four of 144 (38%) men in the dutasteride group were deemed to have progressed at 3 years, compared to 70 of 145 (48%) of controls [3].

Two other randomised trials have investigated the use of anti-androgen therapy in prostate cancer. The ARTS study [4] showed that dutasteride significantly delayed the time to prostate specific antigen (PSA) doubling and disease progression (which included PSA- and non-PSA-related outcomes) compared with placebo after 24 months of treatment (*p* < 0.001).

Conversely, the AVIAS trial [5] was a small-scale phase II randomised controlled trial that showed no benefit to the addition of dutasteride to an intermittent androgen deprivation therapy regimen.

Although it is known that dutasteride is associated with a reduction in the overall prostate volume of 25% after 3-6 months exposure, the mechanism by which prostate cancer prevalence is reduced remains unclear [6].

In order to further explore the mechanisms at work we undertook specific analyses on the diffusion-weighted sequences that were derived from the MAPPED study – a randomised study of dutasteride versus placebo in men with low risk prostate cancer that used multi-parametric Magnetic Resonance Imaging (mpMRI) as an endpoint [7].

## Materials and methods

This study represents a planned analysis from a phase II, randomised, double blind, prospective clinical trial approved by the Hammersmith & Queen Charlotte’s & Chelsea Research Ethics Committee (UK) (09/H0707/84), and the Medicines & Health Regulatory Agency and registered on the European Clinical Trials register (EudraCT 2009-102405-18) [7]. The study was investigator-led, and sponsored by University College London. It was funded through an unrestricted grant by GlaxoSmithKline (GSK). All patients gave written informed consent to participate in this study, and were blinded to treatment allocation.

### Eligibility criteria and study design

Between June 2010 and January 2012, 42 men were recruited, and 40 completed the study; the full study protocol has been published [7]. An initial routine 1.5 T mpMRI for the assessment of prostate cancer showed at least a 0.2 cc lesion at mpMRI (T2-weighted, diffusion-weighted imaging - DWI - or dynamic contrast enhanced - DCE -) for all men, in line with standard guidelines [8]. Eligible men met the UK National Institute for Health and Clinical Excellence (NICE) 2014 active surveillance criteria [9].

Specifically, inclusion criteria for this retrospective analysis were: (1) Gleason 3 + 3 or 3 + 4 prostate cancer based on biopsy within the preceding two years (this biopsy was not standardised and was not part of the trial); (2) PSA ≤ 15 ng/mL; and (3) lesion scoring ≥ 4 at baseline mpMRI, according to the dominant sequence as reported in the Prostate imaging reporting and data system (PI-RADS) version 2 guidelines [10].

From the initial population (*n* = 40) we identified three men with a lesion scoring ≤ 3 at baseline mpMRI according to PI-RADS version 2 guidelines [10], and we therefore excluded them. It is important to stress that all men included in this study did not have any prostate cancer treatment (hormone manipulation, prostatic surgery, and treatment with any 5-alpha reductase inhibitor) in the previous 12 months.

A 3T MR scan including T1- and T2-weighted and DW imaging was performed and, after review by a study radiologist confirming suitability, men were individually randomised (1:1) to placebo or dutasteride using block randomisation with varying block sizes. MpMRI was repeated at 6 months and an exit biopsy was offered to all men, with ten standard cores and additional cores targeted to the MRI lesion using visual registration [11] to include targeting of MR lesions. Prostate Specific Antigen (PSA) was assessed at baseline and 6 months.

### MR imaging technique

All patients underwent MR imaging using a 3T system (Magnetom Verio, Syngo MR B17; Siemens Healthcare, Erlangen, Germany) and a pelvic phased-array coil. All examinations included unenhanced axial, sagittal and coronal turbo spin-echo T2 weighted imaging and axial DWI (*b* values of 0, 100, 800 and 1400 s/mm^2^ used for calculation of ADC map). The parameters on the multi-*b* value DWI were: repetition time 4300 msec, echo time 80 msec, acquisition matrix 126 x 81, field of view 245 mm x 213 mm, slice thickness 5 mm, flip angle 90°, number of averages 6. A dedicated *b* = 1400 s/mm^2^ sequence was obtained using similar parameters but with 16 averages. Dynamic gradient echo sequences were obtained during intravenous injection of 0.1 mmol/kg of body weight of gadoterate meglumine (Dotarem®, Guerbet, Roissy, France) at a rate of 2 mL/s.

The protocol was in line with standard guidelines [8].

### Image analysis

All MR imaging data sets were anonymised prior to retrospective consensus review by two board-certified uro-radiologists (AK and FG, with 10 and 3 years of experience in prostate cancer mpMRI interpretation, respectively) using commercial image viewing software (Osirix ® v. 4.1.2; Geneva, Switzerland). Both readers were unaware of treatment allocation and PSA results and were privy only to the date of the scan. For the purposes of image interpretation, the lesion was defined as a focal area that displayed (1) focal low signal intensity on T2-weighted images, (2) high signal on the DWI (*b* = 1400 s/mm^2^) and (3) focal restricted diffusion on the ADC map and early-peaking enhancement on the dynamic series. The individual lesions were assigned overall scores from 1 to 5 based on the PIRADS v.2 guidelines [10].

### Region of interest (ROI) assessment

Image quality was sufficient to evaluate ADC in all patients. ADC values were obtained from regions of interest (ROI) traced on ADC maps, making reference to the corresponding DWI sequences and the other sequences (T2-weighted and DCE). The largest lesion (index tumour) was chosen for analysis if multiple foci were detected in the same patient.

**Fig. 1:**
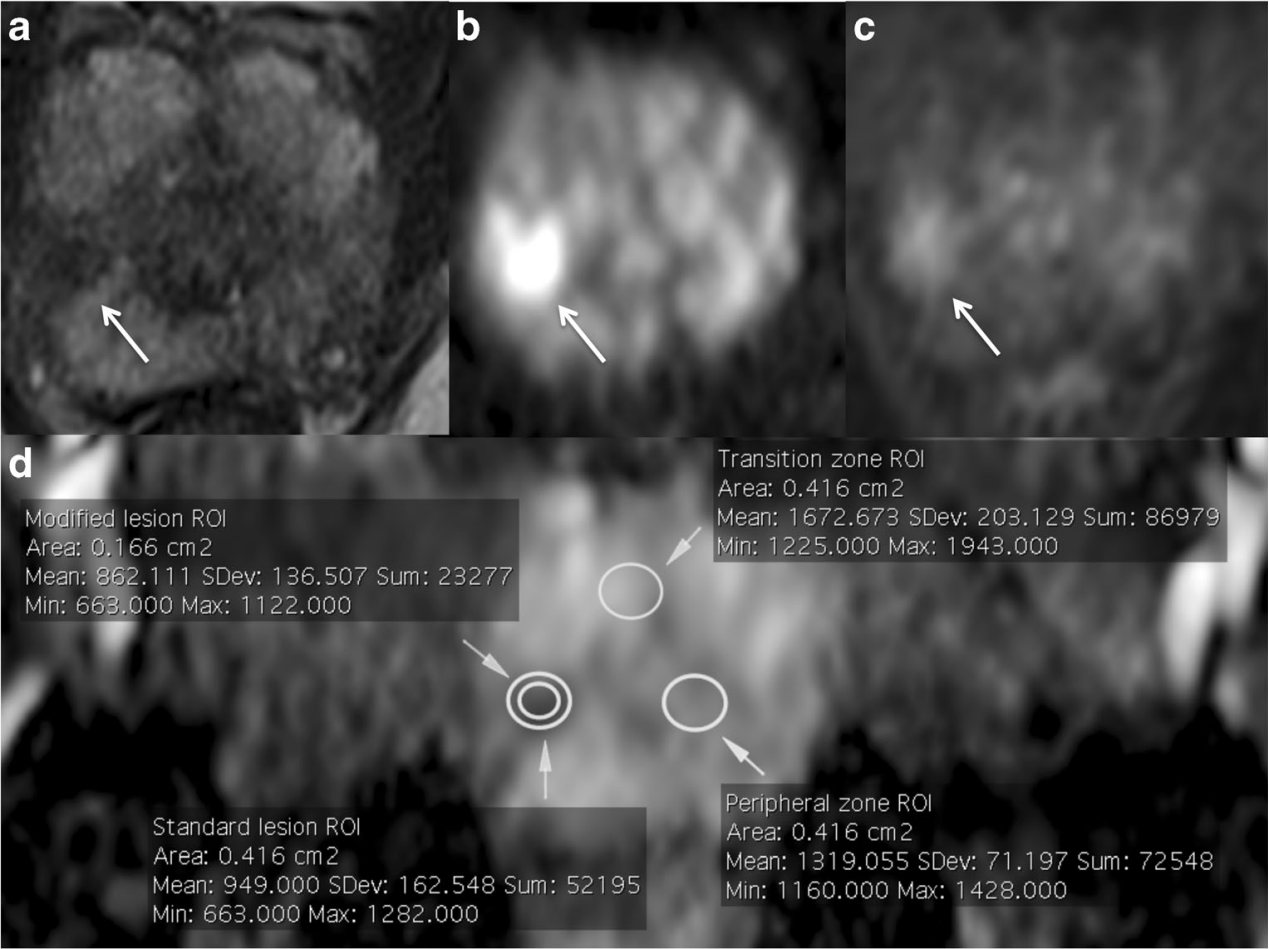
The arrows show a tumour in the right mid-apex peripheral zone of the prostate on T2-weighted (a), diffusion-weighted (b) and dynamic contrast-enhanced (c) imaging, and how all regions of interest (ROIs) were positioned on the same slice of the apparent diffusion coefficient (ADC) map (d) accordingly

**Fig. 2:**
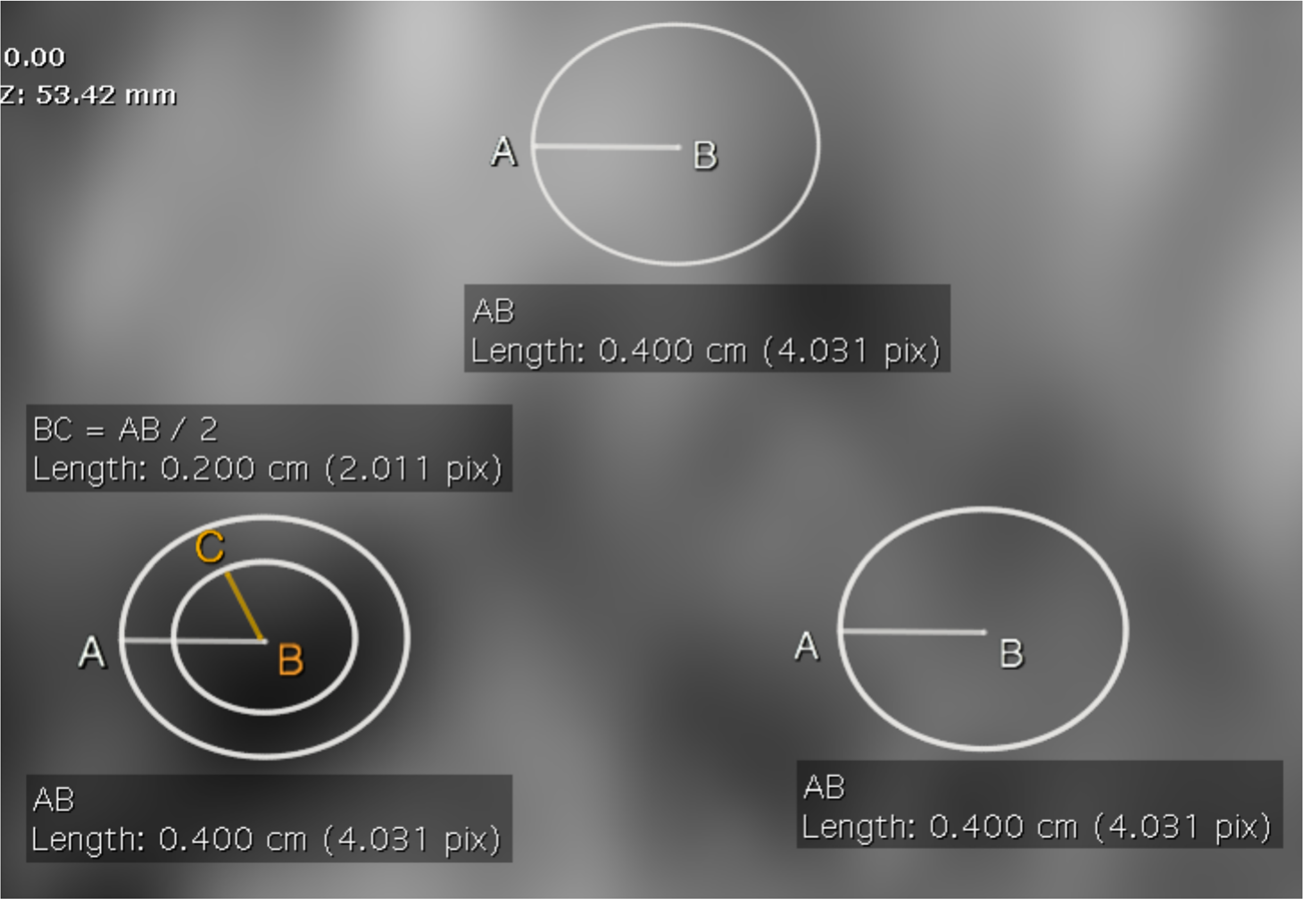
Image from patient reported in Fig. 1 showing our modified method for the calculation of tumour ADC to minimise partial volume effects. A smaller ROI was traced inside the whole tumour ROI, with a diameter (BC) corresponding to the half of the diameter of the greater ROI (AB). The ADC value for the lesion was calculated from this area

On the same slice, the readers also copied and pasted two ROIs of the same size of the whole lesion ROI both in the non-cancerous peripheral (PZ) and transitional (TZ) zone - in mirror position to the lesion (Fig. 1) - and calculated the additional ADC values. Because the ROI of a small lesion (which is a common situation in men on active surveillance) is likely to include elements only partially filling the slice, we used a modified method for the calculation of ADC of the tumour, in order to minimise such partial volume effects. Specifically, a smaller ROI was traced inside the whole lesion ROI, with a diameter corresponding to half of the diameter of the greater ROI, and the mean tumour ADC value was calculated from this area (Fig. 2). The same ROIs were used for the measurement of signal intensity (SI) on the dedicated long *b* (1400 s/mm^2^) DWI sequence.

### Tumour conspicuity

We defined the term conspicuity as the mean ADC of the PZ divided by the mean ADC of the tumour.

### Statistical methods

Clinical and demographic data were reported using descriptive statistics.

Mean and standard deviations were used to summarise continuous variables. Categorical data were expressed by frequencies and percentages.

To detect significant changes in DWI ADC, conspicuity and SI between baseline and 6-month scans, paired T-tests were carried out in the placebo and dutasteride groups. A paired t test was also used to check for differences in the size of the ROIs used to measure these parameters at baseline and 6 months.

To detect a difference between the mean change (either absolute or percentage) of ADC, conspicuity and SI between the two groups (placebo vs dutasteride) we performed unpaired T-tests.

P values less than 0.05 were considered to indicate a significant difference.

All statistical analyses were performed by using SPSS (version 20.0; SPSS, Chicago, Illinois, USA).

## Results

A total of 37 men were analysed in this study (19 in the placebo and 18 in the dutasteride arm, respectively), with a mean age of 65 years (range 49-79). All lesions were visible both at baseline and 6-month scans on DWI, permitting the calculation of tumour ADC values in all men. Of note, 35/37 (95%) suspicious regions evaluated for ADC calculation on mpMRI were concordant for the presence of cancer at entry biopsy. Twenty-eight out of 37 (76%) lesions were also concordant at exit biopsy, 2/37 (5%) were discordant - one in the placebo and one in the dutasteride group - and 7/37 (19%) men declined the exit biopsy - four in the placebo and three in the dutasteride arm.

There were 33/37 (89%) lesions scoring 4 and 4/37 (11%) lesions scoring 5 at baseline mpMRI, according to PI-RADS version 2 [10]. Two lesions in the placebo arm were downgraded from PI-RADS 4 to PI-RADS 3 after 6 months.

Lesion locations were as follows: 33/37 (89%) in the PZ [20/37 (54%) on the right, 12/37 (32%) on the left and 1/37 (3%) midline] and 4/37 (11%) in the TZ.

There was no difference in PSA values between the placebo and the dutasteride group at baseline (6.12 ± 2.20 *vs* 7.14 ± 2.23 ng/mL, *p* = 0.168). There was a significant difference in PSA values between the two arms after 6 months (6.72 ± 2.39 *vs* 4.14 ± 1.65 ng/mL, *p* = 0.001).

Nineteen out of 37 men (51%) had Gleason 3 + 3 and 18/37 (49%) Gleason 3 + 4 at entry biopsy.

Table 1 summarises the mean ROIs measurements at baseline and 6-month scans.

**Table 1:** Mean ROI areas (cm^2^) for each of the two arms at baseline and after 6 months

	Placebo			Dutasteride		
	Baseline MRI	6-month MRI	*p*	Baseline MRI	6-month MRI	*p*
Standard ROI (cm^2^)	0.59 (±0.32)	0.47 (±0.26)	0.088	0.52 (±0.27)	0.55 (±0.26)	0.645
Modified ROI (cm^2^)	0.23 (±0.13)	0.18 (±0.86)	0.111	0.20 (±0.11)	0.21 (±0.11)	0.871

Note - Data are means with standard deviations in parentheses. MRI: magnetic resonance imaging; ROI: region of interest. Paired t test used for comparisons

Table 2 shows mean ADC values, conspicuity and SI from mpMRI at baseline and at the end of the trial (6 months). There were no significant differences over 6 months both for men randomised to placebo or dutasteride for ADC values. A decrease in mean conspicuity and SI over 6 months was observed for men on dutasteride (1.54 *vs* 1.38; p = 0.025) and (56.59 vs 48.99, *p* < 0.01), respectively.

**Table 2:** ADC, conspicuity and signal intensity values for each of the two arms included in the study at baseline and after 6 months

	Placebo			Dutasteride		
	Baseline MRI	6-month MRI	*p*	Baseline MR	6-month MRI	*p*
ADC lesion	0.99 (±0.24)	0.96 (±0.23)	0.301	1.01 (±0.15)	1.08 (±0.20)	0.069
Conspicuity	1.56 (±0.31)	1.67 (±0.34)	0.174	1.54 (±0.26)	1.38 (±0.31)	0.025
ADC TZ	1.46 (±0.18)	1.39 (±0.16)	0.061	1.32 (±0.14)	1.36 (±0.14)	0.307
ADC PZ	1.50 (±0.23)	1.55 (±0.24)	0.446	1.52 (±0.17)	1.45 (±0.21)	0.126
SI lesion	68.05 (±15.96)	71.80 (±13.84)	0.168	56.59 (±15.15)	48.99 (±12.37)	<0.01

Note - Data are means with standard deviations in parentheses. MRI: magnetic resonance imaging; ADC: apparent diffusion coefficient (x 10^-3^ mm^2^/s); TZ: transition zone; PZ: peripheral zone; SI: signal intensity. Paired t test used for comparisons

**Fig. 3:**
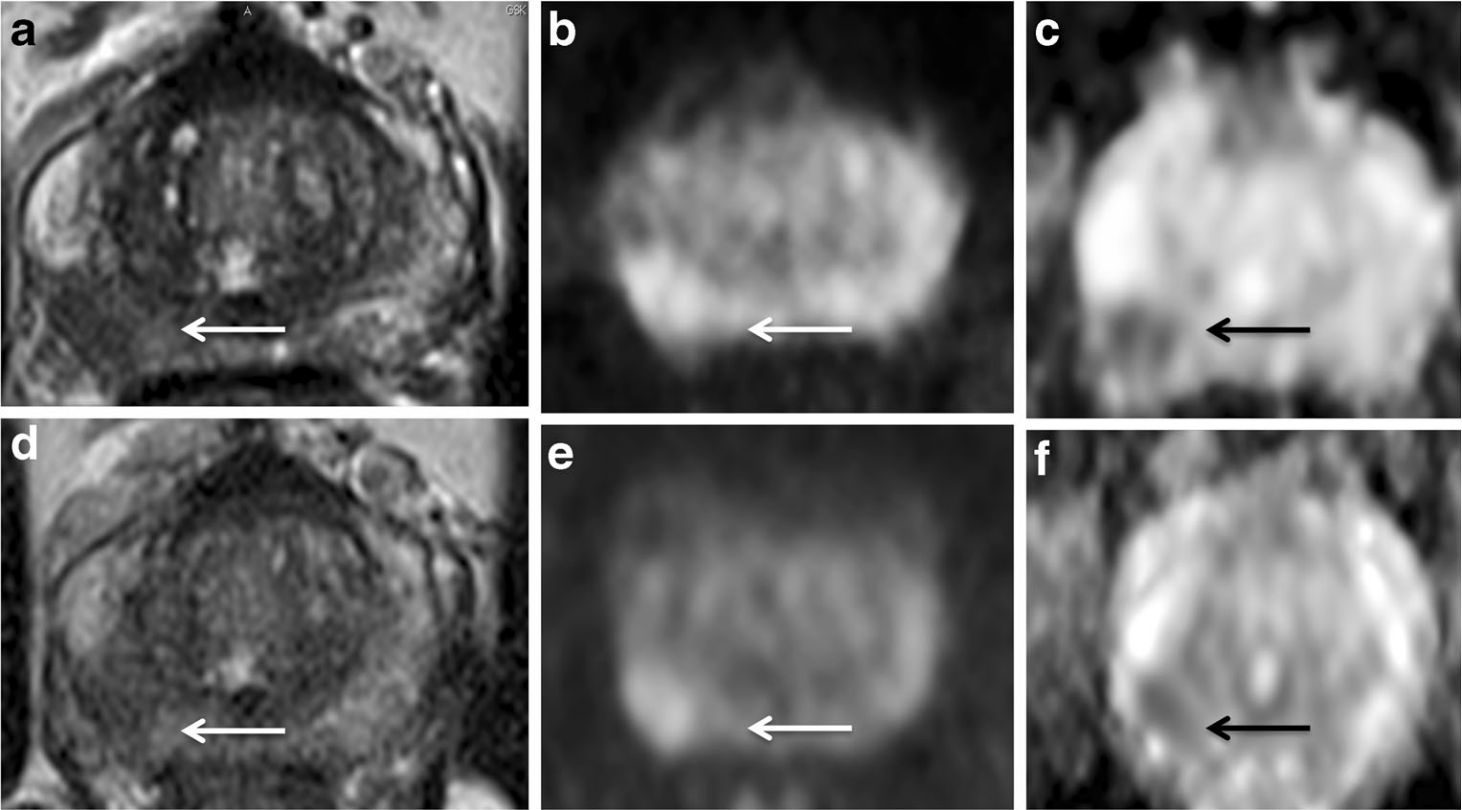
63-year-old man with a presenting PSA of 5.81 ng/mL and Gleason Score 3 + 4. At baseline MRI, the pathological area of decreased signal intensity in the mid-right peripheral (arrow) on the axial T2-weighted image (A) corresponds to the high-signal intensity on the DWI image (B) and low-signal intensity on the ADC map (C), with a reduced ADC value (0.88 x 10^-3^ mm^2^/s). At 6-month MRI (PSA: 2.41 ng/mL) the pathological area is less recognisable (arrow) in all the three scans (D,E,F) and an increase in the ADC value was observed (1.01 x 10^-3^ mm^2^/s). There was a decrease in conspicuity of 11% on the ADC map. This patient was in the dutasteride arm

**Fig. 4:**
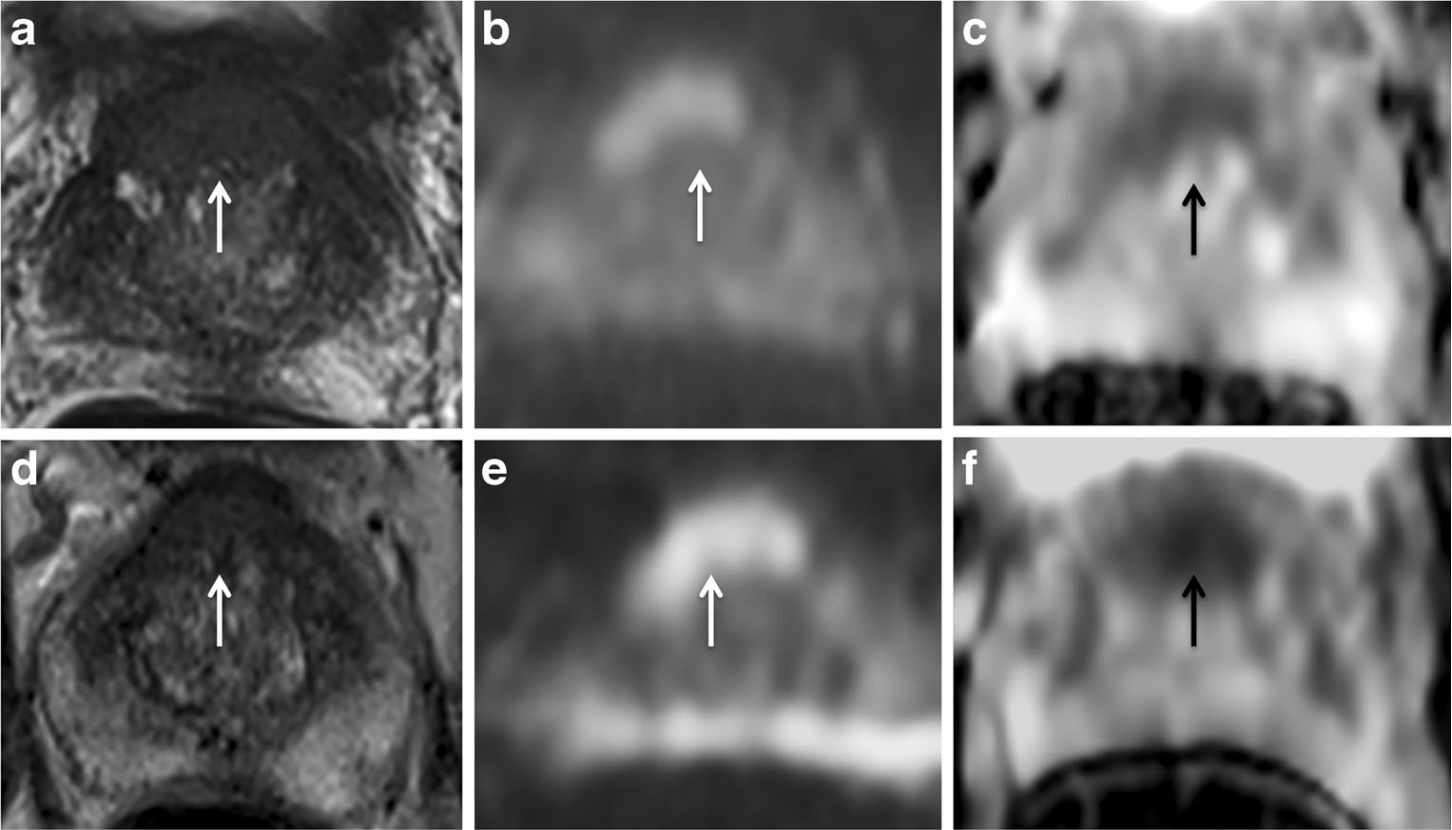
67-year-old man with a presenting PSA of 6.64 ng/mL and Gleason Score 3 + 3. At baseline MRI, the pathological area of decreased signal intensity in the anterior part of the transition zone (arrow) on the axial T2-weighted image (A) corresponds to the high-signal intensity on the DWI image (B) and low-signal intensity on the ADC map (C), with a reduced ADC value (0.87 x 10^-3^ mm^2^/s). At 6-month MRI (PSA: 8.10 ng/mL) the pathological area is still recognisable (arrow) in all the three scans (D,E,F) and a decrease in the mean ADC value was observed (0.63 x 10^-3^ mm^2^/s). There was an increase in conspicuity of 36% on the ADC map. This patient was in the placebo arm

Table 3 reports the difference in absolute values between men taking placebo or dutasteride over 6 months. Significant changes in absolute tumour ADC and conspicuity between the two groups were observed (-0.03 *vs* 0.08, *p* = 0.033) and (0.11 *vs* –0.16, *p* = 0.012), respectively (Fig. 3 and Fig. 4). A significant difference was also noted for ADC values in the TZ (-0.07 vs 0.04, *p* = 0.039) and signal intensity (3.75 vs –5.93, *p* = 0.036), respectively.

**Table 3:** Differences of absolute values over 6 months for ADC, conspicuity and signal intensity for each of the two arms included in the study

	Placebo	Dutasteride	*p*
ADC lesion	- 0.03 (±0.13)	0.08 (±0.17)	0.033
Conspicuity	0.11 (±0.33)	- 0.16 (±0.28)	0.012
ADC TZ	- 0.07 (±0.16)	0.04 (±0.16)	0.039
ADC PZ	0.05 (±0.29)	- 0.07 (±0.19)	0.132
SI lesion	3.75 (±11.38)	- 5.93 (±15.42)	0.036

Note - Data are means with standard deviations in parentheses. MRI: magnetic resonance imaging; ADC: apparent diffusion coefficient (x 10^-3^ mm^2^/s); TZ: transition zone; PZ: peripheral zone; SI: signal intensity. Unpaired t test used for comparison between placebo and dutasteride groups

**Fig. 5:**
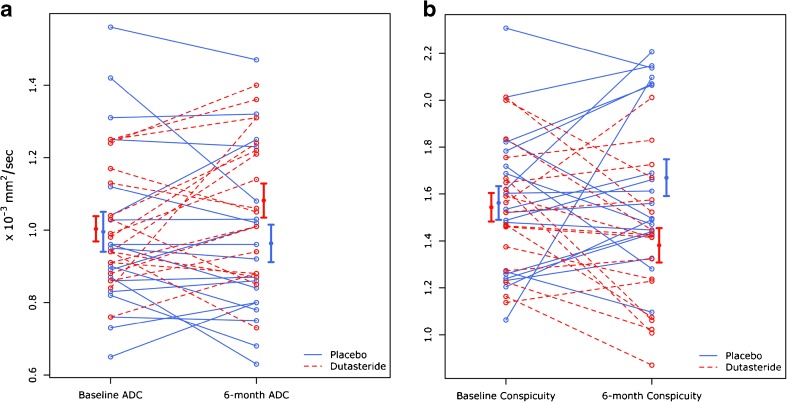
Ladder plots showing tumour ADC (A) and conspicuity (B) changes over 6 months for each of the two arms included in the study. The error bars at 6 months confirm a significant increase in ADC and decrease in conspicuity in men treated with dutasteride (red, dashed line) when compared to men in the placebo arm (blue, continuous line)

Table 4 shows the comparison of the percentage changes between men taking placebo or dutasteride. A significant percentage increase in tumour ADC (8.56% *vs* -2.27%, *p* = 0.048) and a significant decrease in conspicuity (-9.89% *vs* 9.25%, *p* = 0.013) and SI (-6.95% vs 7.93%, *p* = 0.039) were observed in the dutasteride group when compared to the placebo arm (Fig. 5).

**Table 4:** Differences for ADC, conspicuity and signal intensity change over 6 months (expressed as percentage) for each of the two arms included in the study

	Placebo	Dutasteride	*p*
Δ ADC (%)	- 2.27 (±13)	8.56 (±18)	0.048
Δ Conspicuity (%)	9.25 (±26.18)	-9.89 (±17.34)	0.013
Δ SI lesion (%)	7.93 (±0.21)	-6.95 (±0.22)	0.039

Note - Data are means with standard deviations in parentheses. Δ: change (%); ADC: apparent diffusion coefficient; SI: signal intensity. Unpaired t test used for comparison between placebo and dutasteride groups

## Discussion

In summary, men with localised prostate cancer who were randomised to dutasteride exhibited a (1) significant decrease in mean tumour conspicuity, (2) a significant reduction on SI on DWI, and (3) a significant increase in tumour ADC, over a 6-month period compared to the men who were randomised to placebo.

Before considering the clinical and research implications of our findings, it is worth reviewing some of the inevitable methodological issues that were inherent with both the design and conduct of this first ever study to use imaging as a primary endpoint in a study of localised prostate cancer.

The first, and possibly most important, is that the men entered the study largely by means of transrectal ultrasound (TRUS) guided biopsy but exited the study by an image-guided biopsy. As we now know (but were less aware of when the study was conceived) these two methods of assessment yield quite different results given a stable disease status [12]. Fortunately, this aspect of the trial design does not impact on the objectives of our current analysis, which limits itself to the mpMRI-derived lesions.

Second, the cohort is relatively small and, as a result, may not be fully representative of the spectrum of mpMRI-derived lesions in the population. The original MAPPED study was powered to show a 20% difference in tumour volume, and the predetermined end points were met. Clearly, further studies are going to be needed to improve the degree to which we are representative of the disease that we are studying. For instance, peripheral zone tumours were over represented in our cohort, and, as a consequence, transition zone tumours were less common. This might explain our observation of a reduction in ADC within the transition zone of the placebo-treated tumours - something we did not observe in the peripheral zone. The inherent heterogeneity of the transition zone is likely to introduce bias and that will only be countered by increasing the number of subjects assessed.

Finally, the method used for the measurement of ADC may (even with the modified method used in this study) be prone to some edge effects and this is a problem that will affect any study using parametric assessment of small prostate lesions: the possibility that changes in lesion size will change *apparent* quantitative measurements because of edge effects must always be borne in mind.

Despite these methodological issues, we feel that our findings are important both clinically and in terms of our future understanding of prostate cancer progression.

From a clinical perspective our results show that men on dutasteride may be less likely to have their cancer called: reduced ADC (both absolute and relative to surroundings) and high signal on long *b* images are the key elements of tumour detection using DWI in PIRADS 2 [10]. In response, as a minimal standard, radiologists should be made aware of the dutasteride or finasteride exposure to help them interpret the finding on mpMRI. For the future, the architects of PIRADS III or IV may wish to make specific recommendations for men exposed to these agents.

As the exposure to antiandrogen therapy seems to reduce tumour conspicuity when mpMRI are performed to assess for prostate cancer, our findings support the idea that a lower threshold for triggering biopsy might be considered in men taking dutasteride or finasteride. In active surveillance it is possible that these drugs may increase the proportion of small, low grade tumours that are seen poorly or not at all, a factor which might be taken into account in follow-up protocols.

From a research perspective our results are of considerable interest in that they point to a potentially valid, largely reliable and responsive imaging biomarker that may be providing non-invasive information on tumour status. In 2009, Padhani et al. alerted us to the importance (and potential utility) of assessing the effect of a drug (in absolute terms and in direction) on ADC values [13]. Our data provides a preliminary response to this challenge.

Nevertheless, the question, ‘What biological processes is mpMRI revealing to us?’ remains both important and pertinent.

We know from radical prostatectomy studies that were conducted after short-term dutasteride exposure that cellular involution and epithelial shrinkage occurs in benign tissue in addition to the increase in the stromal/epithelial ratio in prostate cancer, suggesting that dutasteride may induce significant phenotypic alterations in both the benign and the neoplastic prostate [14]. It is possible therefore that the increase in ADC that we are observing in dutasteride-treated prostate cancer may be due to a change in the cellular components of the cancer.

The ADC increase that we observed in the dutasteride arm of the study is certainly consistent with previous evidence; an increase in ADC values has been associated when a cytotoxic selective pressure was applied [15]. In addition, antiandrogen treatments (dutasteride being a weaker bedfellow) have been shown to reduce prostatic blood flow, causing nuclear shrinkage, cell vacuolisation, apoptosis and necrosis in prostate cancer [16], resulting in an increase in the ADC values. There is some suggestion that these ADC values, whatever it is they are telling us about tumour status, may have some clinical relevance. De Souza et al. [17] previously showed that the ADC of high or intermediate risk prostate cancer is lower than that of low-risk tumours and suggested that in patients with low-risk, localised disease, tumour ADC may be a useful marker of progression [18].

In conclusion, our results show that dutasteride is associated with an increase in tumour ADC and reduces tumour conspicuity on DWI. This may adversely impact on cancer detection rates in men exposed to 5-alpha reductase inhibitors. As a minimum, radiologists should be made aware of the treatment status of the prostate.

## Abbreviations

DHT: Dihydrotestosterone
LUTS: Lower urinary tract symptoms
ROI: Region of interest
SI: Signal intensity
